# Relationship Between Prognostic Nutritional Index and Stroke‐Associated Pneumonia in Elderly Patients: A Two‐Center Study of Spontaneous Intracerebral Hemorrhage

**DOI:** 10.1002/fsn3.71941

**Published:** 2026-05-23

**Authors:** Gang Wang, Guangyan Li, Wenjun Zhao, JiaYin Wang, Dong Wang, Qunbin Shen, Boru Hou

**Affiliations:** ^1^ Department of Neurosurgery, The Second Hospital & Clinical Medical School Lanzhou University Lanzhou Gansu China; ^2^ Key Lab of Neurology of Gansu Province Lanzhou Gansu China; ^3^ The Second Hospital & Clinical Medical School Lanzhou University Lanzhou Gansu China; ^4^ Department of Ultrasound The Third People's Hospital of Gansu Province Lanzhou Gansu China; ^5^ Department of Neurosurgery The Second Affiliated Hospital of Fujian Medical University Quanzhou Fujian China; ^6^ Department of Neurosurgery The Second Affiliated Clinical Medical College of Fujian Medical University Quanzhou Fujian China

**Keywords:** mediator analysis, prognosis nutrition index, spontaneous intracerebral hemorrhage, stroke‐associated pneumonia

## Abstract

Stroke‐associated pneumonia (SAP) is a common complication in elderly patients with spontaneous intracerebral hemorrhage (sICH), and nutritional‐immune status may influence the risk of infection. The prognostic nutritional index (PNI), which integrates nutritional and immune‐related markers, has been proposed as a potential indicator of clinical outcomes. However, evidence regarding its association with SAP remains limited. This two‐center retrospective study enrolled elderly patients with sICH admitted to Lanzhou University Second Hospital and Fujian Medical University Union Hospital. Patients were grouped according to the occurrence of SAP, and demographic characteristics, laboratory indicators, and imaging data were collected. Multivariate logistic regression analysis was performed to examine the association between PNI and SAP after progressively adjusting for potential confounding factors. Restricted cubic splines (RCS) were used to explore the dose–response relationship. Bayesian mediation analysis was conducted to assess the mediating effect of white blood cell count (WBC) between PNI and SAP, and stratified subgroup analysis was further performed to evaluate the stability of the association. Among 431 patients, the incidence of SAP was 64.0%. Multivariate logistic regression revealed a significant negative relationship between PNI and SAP risk (OR = 0.95, 95% CI: 0.90–1.00). Trend analysis indicated a linear relationship between elevated PNI levels and reduced SAP risk (*p* trend < 0.05), whereas RCS analysis did not reveal significant nonlinear associations. Mediation analysis indicated that WBC partially mediated the relationship between PNI and SAP, with a mediation proportion of 10.14%. Subgroup analysis showed that this association was more pronounced in patients with GCS < 9 and greater blood loss (*p* for interaction < 0.05). These findings suggest that reduced PNI is a significant risk indicator for the development of SAP in elderly patients with sICH, and PNI may influence infection risk partly through its intermediary effect on the inflammatory response.

## Graphical abstract

Low PNI indicates an increased risk of stroke‐associated pneumonia in elderly patients with spontaneous intracerebral hemorrhage. Early assessment of albumin and lymphocytes may help identify high‐risk patients and guide targeted nutritional, respiratory, swallowing, and infection‐prevention strategies.

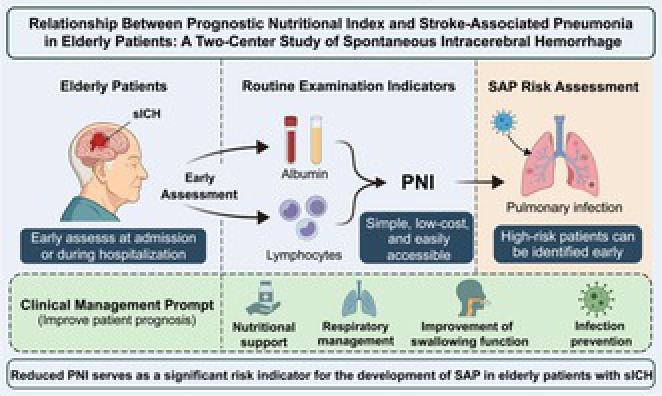

## 1 Introduction

Intracerebral hemorrhage exhibits etiological heterogeneity and is generally classified into spontaneous and secondary types according to its underlying causes (Jain et al. 2021). Spontaneous intracerebral hemorrhage (sICH) accounts for approximately 80% of all cases and is mainly associated with hypertension, cerebral amyloid angiopathy, or unknown causes. Unlike spontaneous intracerebral hemorrhage, secondary intracerebral hemorrhage is usually attributable to identifiable causes, such as cerebral arteriovenous malformations, intracranial aneurysms, and Moyamoya disease (Sarfo et al. 2020). sICH is one of the most devastating forms of stroke, accounting for approximately 10%–15% of all strokes, yet its mortality and disability rates are significantly higher than those of ischemic stroke (Finlayson et al. 2011). Epidemiological data indicate that the 30‐day mortality rate for ICH can reach 35%–50%, with over half of survivors experiencing moderate to severe disability (Roy‐O'Reilly et al. 2017). Elderly patients often present with age‐related vascular changes, impaired immune function, and multiple comorbidities, and are also more likely to use antithrombotic or anticoagulant medications. These factors may increase their risk of perioperative bleeding‐related complications and postoperative infections following spontaneous intracerebral hemorrhage. In recent years, improvements in critical care and neurosurgical interventions have enhanced acute‐phase survival rates for ICH patients. However, infectious complications, particularly stroke‐associated pneumonia (SAP), remain a significant cause of deterioration and mortality (Vyas et al. 2019). Following a stroke, most patients often develop SAP due to aspiration. SAP is particularly common in patients with hemorrhagic stroke, representing one of the most significant infectious complications. Incidence rates reported in various studies range from approximately 20%–40% (Zhao et al. 2024). It not only significantly prolongs hospital stays and increases medical costs but is also closely associated with poor neurological recovery, rebleeding, and long‐term mortality risk (Lv et al. 2023; Abujaber et al. 2025). In a multicenter study of 915 ischemic stroke patients, Li et al. (2023) reported a SAP incidence of 32.1%, with significantly higher 30‐day mortality in SAP patients compared to non‐SAP patients. Similarly, in patients with severe ICH, Zhao et al. (2024) reported a SAP incidence of 21.3%, with an in‐hospital mortality rate of 17.0%. Previous studies also indicate that post‐stroke pneumonia typically occurs within 7 days of onset, peaking on the third day (de Jonge et al. 2022). Therefore, early identification of high‐risk individuals and implementation of intervention measures are crucial for improving patient outcomes.

Currently recognized risk factors for SAP include advanced age, impaired consciousness, massive hemorrhage, dysphagia, and mechanical ventilation (Erfani et al. 2022). In addition to these clinical risk factors, stroke‐induced immunodepression syndrome (SIDS) has been described by Prass et al. (2006) as a rapid and persistent peripheral immunosuppressive state after stroke, characterized by lymphopenia, reduced monocyte activity, Th1/Th2 imbalance, and decreased interferon‐γ production. In ICH patients, SIDS may coexist with neuroinflammatory activation, creating an immune‐inflammatory imbalance that contributes to the development of SAP. However, these factors predominantly represent indicators of disease severity or clinical characteristics that are difficult to intervene upon early. In recent years, as the importance of “systemic status” in stroke prognosis has gained increasing attention, researchers have begun exploring the value of inflammation and nutrition‐related indicators in predicting infection risk (Iddir et al. 2020; Huang et al. 2024). Substantial evidence suggests that malnutrition is closely linked to impaired immune function and represents a significant contributor to increased susceptibility to infection (Ilkowski et al. 2025; Vemuri et al. 2018). This occurs because malnutrition causes “omnidirectional damage” to the immune system by cutting off essential raw material supplies, disrupting tissue structures, and interfering with signal transduction. Elderly ICH patients commonly exhibit malnutrition, excessive inflammatory responses, and immune dysregulation. This “inflammation‐malnutrition imbalance” state may constitute a critical pathological basis for SAP (Jhang et al. 2024).

The prognostic nutritional index (PNI) comprises albumin and lymphocyte counts, providing a comprehensive reflection of systemic inflammation and nutritional status. Albumin reflects the body's nutritional and synthetic capacity while also correlating with inflammatory responses; lymphocytes represent cellular immune function. The combination of these two components facilitates a holistic assessment of the body's immune‐nutritional status (Sun et al. 2025). Initially developed for surgical risk assessment, recent studies have demonstrated PNI's significant prognostic value across cardiovascular diseases, malignancies, and infectious diseases (Ding et al. 2022; Xu et al. 2021). In stroke research, PNI has demonstrated broad application potential. Nergiz and Ozturk (2023) observed significantly lower PNI levels in the infection group versus the non‐infection group among acute ischemic stroke patients (34 vs. 124 cases, *p* < 0.001), with a 21% infection rate among those with PNI < 350. Li et al. (2023) further demonstrated that among 915 ischemic stroke patients, moderate‐to‐severe malnutrition, defined as a CONUT score of 5–12, significantly increased the risk of SSI, with an adjusted odds ratio (OR) of 6.941. However, existing research has primarily focused on ischemic stroke or general stroke populations, while studies on elderly individuals with spontaneous intracerebral hemorrhage remain limited. Elderly patients are characterized by immune aging, reduced protein synthesis capacity, and a higher prevalence of chronic comorbidities. Consequently, the relationship between changes in their PNI levels and infection risk may differ from that observed in other types of stroke. More importantly, the existence of an inflammatory pathway‐mediated interaction (such as those of leukocyte changes) between PNI and SAP remains unverified. This uncertainty renders the role of PNI in the pathogenesis of sICH‐SAP unclear.

Based on this, the present study systematically evaluated the relationship between PNI levels and the risk of developing SAP in elderly patients with sICH across multiple centers. Furthermore, mediation analysis was employed to explore the potential bridging role of inflammatory responses between these two factors. By validating the clinical value of PNI in predicting SAP among elderly sICH patients, this research aims to provide evidence‐based support for the early identification of high‐risk patients, the development of personalized nutritional interventions, and the formulation of infection prevention strategies.

## 2 Materials and Methods

### 2.1 Study Design and Study Population

This study is a Two‐Center, retrospective investigation designed to analyze the relationship among clinical characteristics, relevant indicators, and outcomes in elderly patients with intracerebral hemorrhage. The study received ethical approval from the Ethics Committees of the Second Affiliated Hospital of Lanzhou University (Approval No.: 2025A‐1073) and the Second Affiliated Hospital of Fujian Medical University (Approval No.: 2024‐016), and strictly adhered to the ethical principles outlined in the Declaration of Helsinki. Given that all data utilized in the study were de‐identified, with patient identities completely anonymized, the Ethics Committees waived the requirement of informed consent.

**FIGURE 1:**
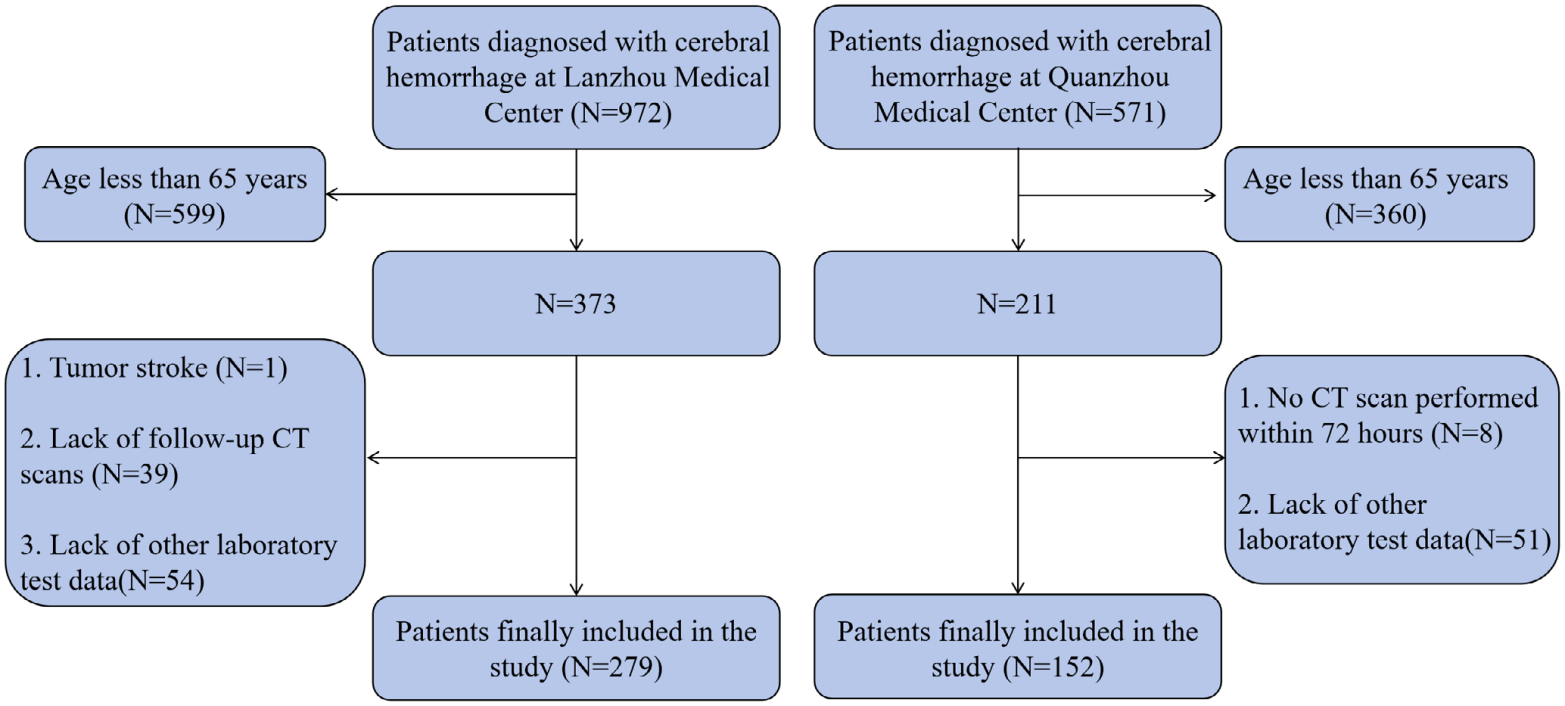
Flowchart for screening elderly patients with cerebral hemorrhage.

The study data were derived from two Grade A tertiary teaching hospitals in China. A total of 1543 patients with intracerebral hemorrhage admitted to Lanzhou University Second Affiliated Hospital between March 2022 and December 2023, and those admitted to Fujian Medical University Second Affiliated Hospital between January 2014 and August 2023, were included. All patients received a definitive diagnosis of intracerebral hemorrhage via cranial computed tomography (CT). Diagnoses were confirmed by qualified neurologists or neurosurgeons to ensure accuracy and consistency. Exclusion criteria include: (1) age under 65 years (Sabharwal et al. 2015); (2) primary intraventricular hemorrhage, or multiple hemorrhagic foci rendering accurate hematoma volume assessment impossible; (3) missing critical clinical information, such as essential laboratory test data; (4) patients who did not undergo cranial and chest CT scans within 72 h of onset; (5) patients lost to follow‐up or who declined study participation; (6) patients with pre‐existing pulmonary infection before intracerebral hemorrhage; and (7) Patients with secondary intracerebral hemorrhage due to cerebral arteriovenous malformation, Moyamoya disease, traumatic brain injury, or hemorrhagic conversion of ischemic stroke. Upon admission, all patients underwent comprehensive assessment and standardized treatment by specialized neurologists according to the “Chinese Stroke Association guidelines for clinical management of cerebrovascular disorders” (Cao et al. 2020). Based on inclusion and exclusion criteria, a total of 431 patients were ultimately enrolled. The screening process for study participants is detailed in Figure 1.

### 2.2 Baseline Characteristics

Each research center conducted data collection through its team members, who received standardized training and utilized their respective hospitals' electronic medical record systems. Before statistical analysis, the research team anonymized all data by removing patient identifiers to ensure participant privacy and confidentiality. The collected clinical data included: basic demographic information (such as age and gender), lifestyle factors (smoking and alcohol consumption), past medical history and medication history (including hypertension, diabetes, and use of anticoagulants or antiplatelet agents), as well as initial clinical data such as the Glasgow Coma Scale (GCS) and body temperature upon admission. All patients underwent venous blood sampling after an 8–12‐h fast within the first 24 h of admission. Laboratory tests included routine blood and biochemical parameters such as white blood cell count, neutrophil count, lymphocyte count, albumin, and blood glucose. Additionally, the research team utilized 3D Slicer software to segment hematomas in imaging data from intracerebral hemorrhage patients and calculated hematoma volume accordingly. Simultaneously, the location of the hematoma, the side of the hemorrhage, and the midline shift were recorded to further optimize the assessment of imaging characteristics. Surgical treatment plans were determined by neurosurgeons based on the patient's specific clinical conditions and current relevant treatment guidelines for sICH.

### 2.3 Exposure Variable Assessment

PNI is commonly used to comprehensively evaluate a patient's inflammatory response, immune function, and nutritional status. This study employs PNI as an indicator of the baseline nutritional and immune status in patients with intracerebral hemorrhage. The PNI is calculated as follows: 5 × lymphocyte count (10^9^/L) + serum albumin level (g/L) (Gp et al. 1980).

### 2.4 Evaluation of Outcome Variables

The primary outcome measure of this study was whether patients developed SAP following sICH (Smith et al. 2015). According to the expert consensus on SAP, SAP refers to lower respiratory tract infections occurring within 7 days after stroke onset. Diagnosis is based on clinical manifestations (such as fever, cough, purulent sputum, altered bronchial sounds, or impaired gas exchange) and laboratory indicators (such as white blood cells), supported by characteristic radiographic abnormalities (such as chest x‐ray, CT, and MRI).

### 2.5 Statistical Analysis

Descriptive analysis characterized continuous variables using means with standard deviation (SD) and categorical variables using frequency distributions with proportional percentages. For intergroup comparisons, categorical variables were compared using Pearson's chi‐square tests, while continuous variables were analyzed with either one‐way analysis of variance (ANOVA) or Kruskal–Wallis rank sum tests, depending on the normality of their distribution. Multivariate logistic regression models were employed to examine the association between PNI and SAP development, with progressive adjustment for confounding factors at three levels. Model 1 served as the unadjusted baseline model, including only PNI as the independent variable. Model 2 incorporated core demographic confounders: age, sex, smoking, alcohol consumption, hypertension, diabetes history, and relevant medication use. Model 3 was further adjusted for all baseline variables. In trend analysis, PNI was modeled both as a continuous variable and as a categorical variable grouped by quartiles. Building upon the three models described above, restricted cubic spline (RCS) analysis was further employed to explore potential nonlinear associations between PNI and the risk of developing SAP. Mediation analysis employed Bayesian methods, modeled using the “mediation” package in R software, to quantify the proportion of the association between PNI and SAP mediated by white blood cell count (WBC). The direct effect of PNI on SAP and the indirect effect mediated through WBC were estimated using 1000 MCMC iterations to estimate the posterior distributions. To further investigate the association between PNI and SAP risk, subgroup analyses were conducted to assess the potential modifying effects of key demographic characteristics, clinical parameters, and bleeding‐related factors on this association. Stratified analyses facilitated the identification of specific subgroups with varying sensitivities to the PNI‐SAP relationship, thereby revealing potential interactions between PNI and other variables.

## 3 Results

### 3.1 Participant Demographics and Clinical Profile

Among the initial 1543 enrolled patients, 431 elderly patients aged 65 years or older were ultimately selected after rigorous screening to form the study cohort. Their overall prevalence rate for SAP was 64%. The mean age of the subjects was 71.41 years with a SD of 5.29 years, and males accounted for 55.91% of the total cohort. In this cohort, 17.4% of patients had a history of smoking, and 11.37% had a history of alcohol consumption (Table S1). Patients were divided into four quartiles (Q1–Q4) based on their PNI levels. Nutritional and inflammation‐related indicators exhibited a dose‐dependent increase as PNI levels rose across quartiles (Table 1). Overall, patients with higher PNI levels demonstrated relatively better systemic health. Specifically, significant relationships were observed between PNI quartiles and various hematopoietic and coagulation markers, such as Hemoglobin (Q1: 135.58 vs. Q4: 148.61) and Platelets (Q1: 176.72 vs. Q4: 218.16) (both *p* < 0.001). Notably, the prevalence of SAP decreased significantly across quartiles (Q1: 74.07%, Q4: 55.05%; *p* = 0.031), highlighting a potential association between higher PNI levels and SAP.

**TABLE 1:** Baseline characteristics of PNI quartiles and SAP.

PNI	Q1	Q2	Q3	Q4	*p*
	*N* = 108	*N* = 105	*N* = 109	*N* = 109	
Age (year)	71.72 ± 5.39	72.06 ± 5.72	71.02 ± 4.57	70.86 ± 5.40	0.344
Temperature (°C)	36.79 ± 0.52	36.73 ± 0.43	36.52 ± 3.23	36.75 ± 0.54	0.481
Leucocyte (10^9^)	10.28 ± 4.45	9.30 ± 3.71	10.22 ± 4.14	10.54 ± 6.15	0.33
Hemoglobin (g/L)	135.58 ± 23.78	138.22 ± 19.75	144.83 ± 21.11	148.61 ± 23.06	< 0.001
Platelets (10^9^)	176.72 ± 78.71	186.18 ± 80.25	183.49 ± 73.64	218.16 ± 103.86	< 0.001
Glucose (mmol/L)	8.08 ± 3.13	7.66 ± 2.74	7.81 ± 2.47	8.22 ± 2.84	0.459
PT (s)	12.97 ± 9.75	12.02 ± 3.28	12.67 ± 4.73	11.81 ± 2.26	0.203
INR	1.05 ± 0.16	1.03 ± 0.29	1.09 ± 0.44	1.00 ± 0.10	0.186
GCS	10.43 ± 3.26	10.72 ± 3.18	10.39 ± 3.31	10.68 ± 3.89	0.701
APTT (s)	26.01 ± 5.78	25.93 ± 5.49	26.70 ± 5.67	26.26 ± 6.90	0.777
Midline shift (mm)	3.02 ± 4.19	1.80 ± 3.02	2.03 ± 3.54	2.28 ± 3.81	0.169
Bleeding volume (mL)	27.29 ± 22.01	26.15 ± 22.32	24.93 ± 20.21	26.20 ± 25.72	0.788
Sex, %					0.105
Female	38 (35.19%)	49 (46.67%)	47 (43.12%)	56 (51.38%)	
Male	70 (64.81%)	56 (53.33%)	62 (56.88%)	53 (48.62%)	
History of hypertension, %					0.078
No	19 (17.59%)	34 (32.38%)	32 (29.36%)	31 (28.44%)	
Yes	89 (82.41%)	71 (67.62%)	77 (70.64%)	78 (71.56%)	
History of diabetes, %					0.251
No	85 (78.70%)	93 (88.57%)	91 (83.49%)	88 (80.73%)	
Yes	23 (21.30%)	12 (11.43%)	18 (16.51%)	21 (19.27%)	
Smoking history, %					0.608
No	86 (79.63%)	89 (84.76%)	88 (80.73%)	93 (85.32%)	
Yes	22 (20.37%)	16 (15.24%)	21 (19.27%)	16 (14.68%)	
Drinking history, %					0.352
No	97 (89.81%)	88 (83.81%)	98 (89.91%)	99 (90.83%)	
Yes	11 (10.19%)	17 (16.19%)	11 (10.09%)	10 (9.17%)	
History of anticoagulant or antiplatelet drug use, %					0.933
No	97 (89.81%)	97 (92.38%)	99 (90.83%)	99 (90.83%)	
Yes	11 (10.19%)	8 (7.62%)	10 (9.17%)	10 (9.17%)	
Bleeding side, %					0.802
Bilateral	0 (0.00%)	0 (0.00%)	0 (0.00%)	1 (0.92%)	
Left	51 (47.22%)	51 (48.57%)	52 (47.71%)	53 (48.62%)	
Right	57 (52.78%)	54 (51.43%)	57 (52.29%)	55 (50.46%)	
Bleeding location, %					0.797
Basal ganglia	57 (52.78%)	54 (51.43%)	56 (51.38%)	50 (45.87%)	
Cerebral cortex	21 (19.44%)	22 (20.95%)	23 (21.10%)	19 (17.43%)	
Cerebellum	11 (10.19%)	8 (7.62%)	9 (8.26%)	11 (10.09%)	
Brainstem	2 (1.85%)	1 (0.95%)	2 (1.83%)	3 (2.75%)	
Thalamus	15 (13.89%)	14 (13.33%)	12 (11.01%)	14 (12.84%)	
Others	2 (1.85%)	6 (5.71%)	7 (6.42%)	12 (11.01%)	
Surgical treatment, %					0.187
No	56 (51.85%)	68 (64.76%)	65 (59.63%)	70 (64.22%)	
Yes	52 (48.15%)	37 (35.24%)	44 (40.37%)	39 (35.78%)	
SAP, %					0.031
No	28 (25.93%)	36 (34.29%)	36 (33.03%)	49 (44.95%)	
Yes	80 (74.07%)	69 (65.71%)	73 (66.97%)	60 (55.05%)	

### 3.2 Intergroup Differences in SAP Prevalence

To investigate the relationship between PNI and SAP occurrence, we compared the clinical characteristics between the SAP group and the non‐SAP group (Table S1). Results showed that the PNI level in the SAP group was significantly lower than that in the non‐SAP group (*p* = 0.012), suggesting that a poorer nutritional and immunological status may be associated with SAP development. Additionally, patients in the SAP group exhibited significantly higher white blood cell counts, blood glucose levels, blood loss, and midline shift compared to the non‐SAP group, while GCS scores and hemoglobin levels were significantly lower (*p* < 0.001). This indicates stronger inflammatory responses, poorer neurological function, and a more severe overall condition in SAP patients. Furthermore, the proportion of patients undergoing surgical intervention was significantly higher in the SAP group (*p* < 0.001).

### 3.3 Association Between PNI and SAP Prevalence

**FIGURE 2:**
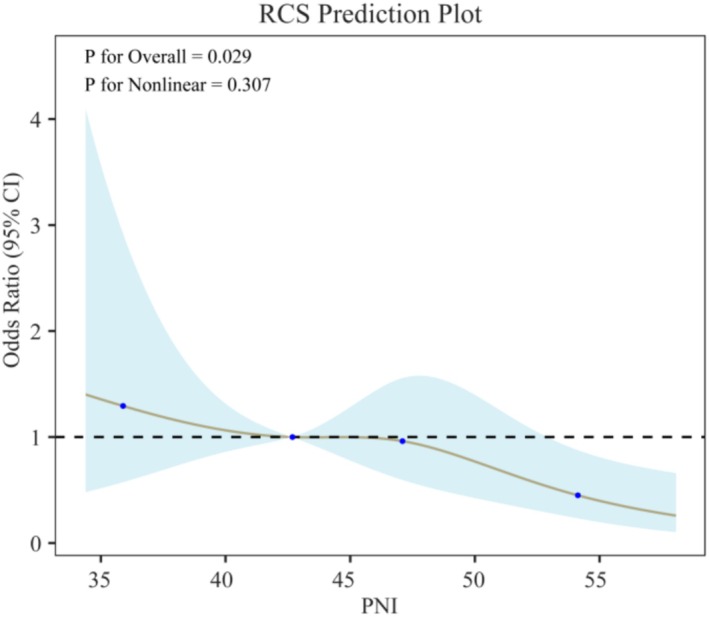
Dose–response relationship between PNI and SAP.

Multivariate logistic regression revealed a robust association between PNI and SAP across all three models. In the crude model, a unit increase in PNI was associated with a 4% reduction in the likelihood of developing SAP (OR = 0.96, 95% CI: 0.93, 0.99) (Table 2). Even in the fully adjusted model (Model 3), a negative association between PNI and SAP remained (OR = 0.95). After further categorizing PNI levels, dose–response trends remained significant across all models (*p* for trend < 0.05), underscoring the strength of the association between elevated PNI and reduced SAP risk. Additionally, RCS analysis was employed to examine the dose–response relationship between PNI and SAP development. Figure 2 shows that when PNI values are low, the ratio increases, suggesting that a decrease in PNI may elevate the risk of SAP. This relationship is statistically significant (*p* for overall = 0.029). Additionally, the *p* value for the nonlinear portion is 0.307, indicating that the relationship between PNI and SAP risk does not exhibit a significant nonlinear pattern. This supports the overall linear trend observed.

**TABLE 2:** Relationship between PNI and SAP.

PNI	SAP		
	Model 1	Model 2	Model 3
	OR (95% CI)	OR (95% CI)	OR (95% CI)
Continuous	0.96 (0.93, 0.99)	0.96 (0.93, 0.99)	0.95 (0.90, 1.00)
*Categories*			
Q1	Reference	Reference	Reference
Q2	0.67 (0.37, 1.21)	0.68 (0.37, 1.24)	0.85 (0.42, 1.71)
Q3	0.71 (0.39, 1.28)	0.73 (0.40, 1.33)	0.89 (0.44, 1.81)
Q4	0.43 (0.24, 0.76)	0.43 (0.24, 0.77)	0.43 (0.20, 0.92)
*p* for trend	0.0056	0.0073	0.0463

*Note:* Model 1: no covariates were adjusted.

Model 2: the following covariates were adjusted: age, sex, history of hypertension, history of diabetes, smoking history, drinking history, and history of anticoagulant or antiplatelet drug use.

Model 3: the following covariates were adjusted: age, temperature, leucocyte, hemoglobin, platelets, glucose, PT, INR, GCS, APTT, midline shift, bleeding volume, sex, history of hypertension, history of diabetes, smoking history, drinking history, history of anticoagulant or antiplatelet drug use, bleeding side, bleeding location, and surgical treatment.

### 3.4 Mediation Analysis

**FIGURE 3:**
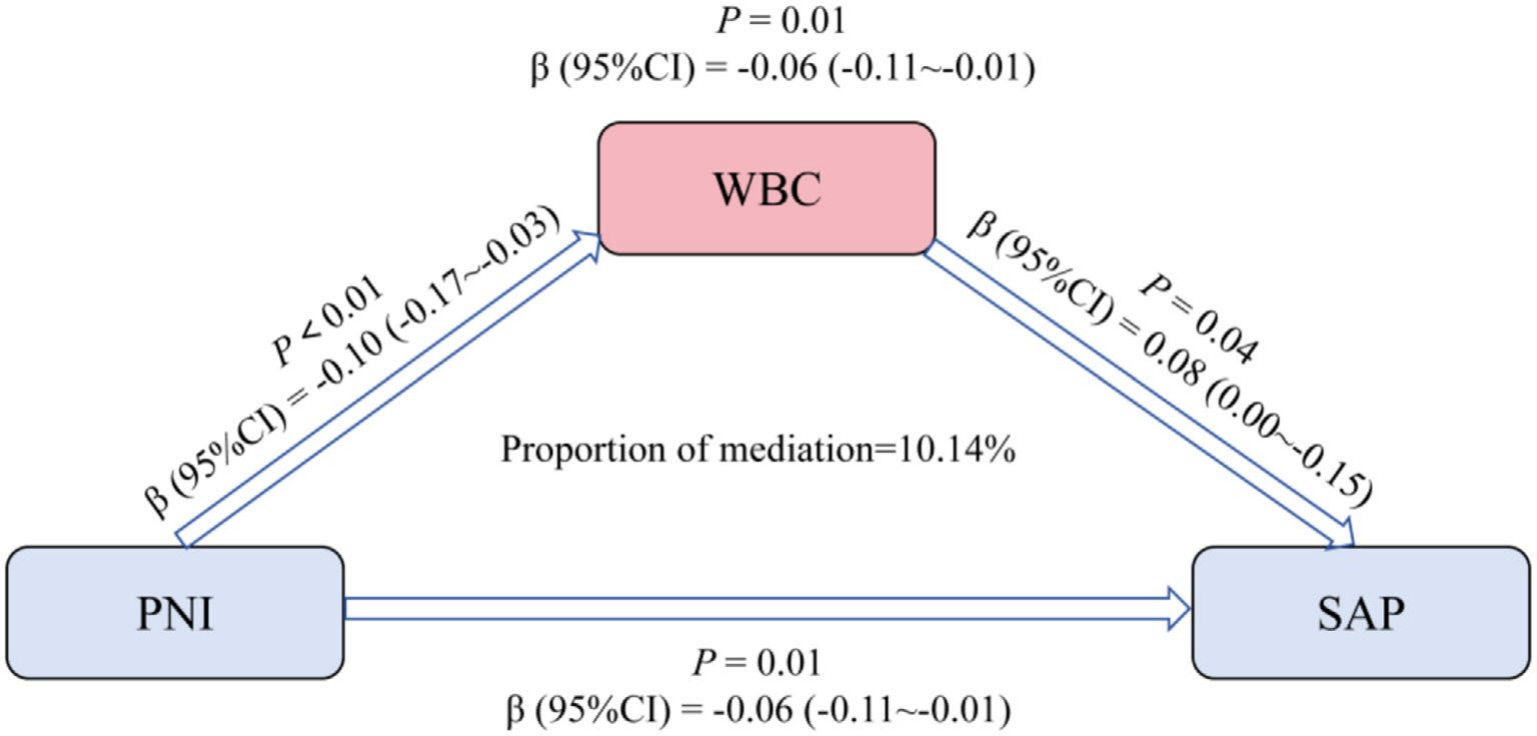
Results of the mediation analysis of inflammatory cells on the relationship between PNI and SAP.

Mediation analysis results indicate that WBC partially mediates the association between PNI and SAP (Figure 3). Specifically, the mediating effect of WBC accounted for 10.14% of the total effect. That is, PNI indirectly influences the risk of SAP by regulating WBC levels. Furthermore, after adjusting for WBC, the direct effect of PNI on SAP remained significant (*p* < 0.05), suggesting that PNI exerts a direct effect on SAP pathogenesis and may also partially act through mediating the inflammatory response (WBC).

### 3.5 Subgroup Analysis

**FIGURE 4:**
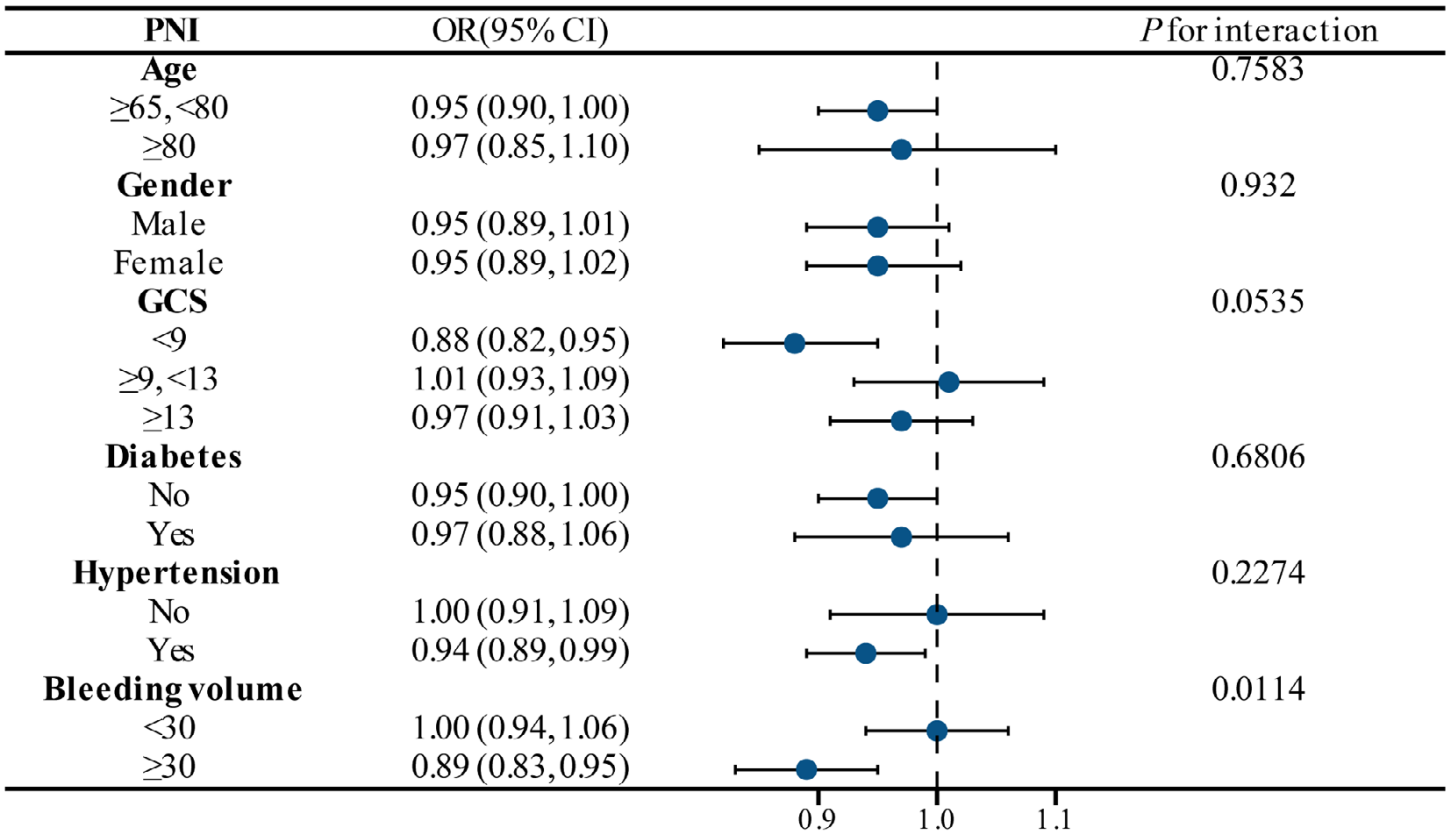
Subgroup analysis of the association between PNI and SAP risk. The dashed line indicates OR = 1. The left side of the dashed line represents protective factors, while the right side indicates risk factors. The interaction *p* value is used to assess the heterogeneity of PNI effects across subgroups.

To further investigate the stability of the association between PNI and SAP, we conducted interaction analyses across multiple clinical subgroups (Figure 4). Results showed that elevated PNI was associated with a trend toward reduced SAP risk in most subgroups, with statistical significance observed in some subgroups. Specifically, among critically ill patients with GCS scores < 9, PNI showed a significant negative relationship with SAP (OR = 0.88, 95% CI: 0.82–0.95), suggesting that PNI may possess stronger predictive power in populations with severe neurological impairment. At the same time, in patients with large bleeding loss, PNI significantly reduced the risk of SAP (OR = 0.89, 95% CI: 0.83–0.95), and the interaction in this subgroup was statistically significant (*p* for interaction = 0.0114), indicating that PNI demonstrates greater discriminatory power in populations with higher hemorrhagic burden. No significant interaction effects were observed in other subgroups such as age, gender, hypertension, or diabetes, suggesting that PNI maintains applicability and stability across different populations.

## 4 Discussion

This two‐center retrospective analysis systematically evaluated the association between PNI and the risk of SAP in elderly patients with sICH. Results demonstrated a significant negative relationship between PNI and SAP incidence, indicating that lower PNI levels were associated with a higher risk of SAP. This association remained stable in multivariate regression and fully adjusted models, and dose–response analysis further confirmed a linear trend between the two factors. Mediation analysis indicated that white blood cell count partially mediated the association between PNI and SAP, which accounted for approximately 10.14% of the total effect. This suggests PNI may indirectly influence pulmonary infection risk through inflammatory pathways. Additionally, subgroup analysis revealed a stronger association in critically ill patients (GCS < 9) and those with larger hemorrhage volumes, indicating enhanced predictive utility of PNI in more severely ill populations.

SAP is one of the most common infectious complications following sICH, with an incidence rate as high as 20%–40%. It significantly increases patients' mortality, disability rates, and length of hospital stay (Finlayson et al. 2011). Elderly patients are at particularly high risk of SAP due to impaired immune function, compromised swallowing reflexes, malnutrition, and multiple chronic disease burdens (Sporns et al. 2017). Previous predictive models have primarily relied on disease severity indicators (GCS score, hemorrhage volume, mechanical ventilation), which, while reflecting clinical status, are difficult to utilize for early intervention (Panchal et al. 2015; Lehmann et al. 2021). Therefore, identifying a simple, quantifiable, and early‐stage predictor that reflects the patient's systemic condition is crucial for improving infection control in elderly ICH patients. This study found that each unit increase in PNI was associated with approximately a 5% reduction in SAP risk, a finding that remained stable after multivariable adjustment. These results indicate that PNI not only reflects nutritional status but also comprehensively reflects the body's immune reserve and inflammatory response capacity. Consequently, it can identify high‐risk individuals for infection early in hospitalization, providing a scientific basis for individualized prevention and treatment strategies. Compared with previous studies, the findings of this research demonstrate innovation in both study subjects and mechanism exploration. Prior research has primarily focused on ischemic stroke populations. Nergiz and Ozturk (2023) found that low PNI significantly increased infection risk in acute ischemic stroke patients; Li et al.'s (2023) multicenter study further indicated that moderate‐to‐severe malnutrition was significantly associated with post‐stroke pneumonia incidence. However, evidence regarding intracerebral hemorrhage patients remains relatively limited. Patients with ICH not only experience reduced nutritional intake but also face the combined effects of stress responses, hematoma compression, and secondary neuroinflammation, resulting in a more pronounced immunosuppressive state (Peng et al. 2019). Therefore, the relationship between PNI and SAP holds greater clinical significance in sICH patients. Zhao et al. reported that nutritional and inflammatory markers have some value in predicting post‐ICH pneumonia, but they did not include PNI in their model nor explore the mediating role of inflammatory responses (Zhao et al. 2024). This study is the first to demonstrate the independent association between PNI and SAP risk in a Two‐Center cohort of elderly sICH patients. Through mediation analysis, it reveals the mediating role of the inflammatory response, providing new evidence for understanding the mechanism of “nutritional‐immune‐inflammatory imbalance” in the pathogenesis of infection.

From a biological mechanism perspective, PNI comprises serum albumin and peripheral lymphocyte count, thus comprehensively reflecting an individual's nutritional and immune status (Yu et al. 2023). Albumin not only maintains colloid osmotic pressure and participates in molecular transport and antioxidant reactions, but also functions as a negative acute‐phase reactant. Decreased albumin levels often indicate systemic inflammatory activation and impaired hepatic synthetic function (Lima et al. 2022). When serum albumin falls below 35 g/L, infection risk increases approximately 1.8‐fold (Lu et al. 2020). Under hypoalbuminemic conditions, antioxidant defenses weaken, reactive oxygen species (ROS) clearance is impaired, and proinflammatory factors such as IL‐6, TNF‐α, and CRP levels rise, leading to increased capillary permeability and delayed tissue repair (Cancello et al. 2023), thereby compromising the alveolar epithelial barrier and increasing susceptibility to pathogen colonization and infection (Chen et al. 2025). Conversely, lymphocyte counts reflect cellular immune function, and reductions typically indicate immunosuppression and impaired defensive capacity (Cao et al. 2016). Previous studies have shown that frailty is a recognized syndrome in older adults, characterized by reduced physiological reserve, malnutrition, and impaired immune function (Hoogendijk et al. 2019; Evans et al. 2022). Because PNI is composed of serum albumin and lymphocyte count, it may partially reflect the nutritional and immunological deficits commonly seen in frail older patients. Studies demonstrate that peripheral T lymphocyte and NK cell counts may decrease by 30%–50% within 24–72 h after stroke onset, indicating a significant immunosuppressive response (Hoffmann et al. 2017). This reaction is termed SIDS, representing a key pathological basis for post‐stroke infections (Liu et al. 2018). The high‐stress state following intracerebral hemorrhage activates the sympathetic nervous system and the hypothalamic–pituitary–adrenal (HPA) axis, leading to the massive release of norepinephrine and glucocorticoids. This further suppresses peripheral immune cell proliferation and cytokine secretion, establishing a persistent state of immune hyporesponsiveness mediated by neuro‐immune interactions (Lehmann et al. 2016). Concurrently, intestinal barrier dysfunction and translocation of gut microbiota are observed post‐stroke. Enteric bacteria entering the circulation can induce systemic inflammatory responses, further exacerbating pulmonary susceptibility (Yin et al. 2021). The combination of nutritional deficiency with this inflammatory and immune imbalance traps patients in a pathogenic vicious “inflammation‐immunity‐metabolism” cycle. The partial mediating effect of white blood cell count in this study further supports the hypothesis that PNI may modulate the risk of infection through inflammatory pathways. Reduced PNI not only reflects inadequate nutritional reserves but also indicates concurrent systemic inflammatory activation alongside immunosuppression, serving as a comprehensive marker of infection susceptibility following intracerebral hemorrhage.

The clinical application potential of PNI warrants attention. As a parameter obtainable through routine hematological testing, PNI is simple to calculate, has strong operational feasibility, features high operational feasibility, and is low‐cost, making it suitable for early risk screening and dynamic monitoring in elderly sICH patients. For patients with low PNI, comprehensive interventions should be initiated early during hospitalization. These include nutritional support, respiratory management, swallowing function improvement, and infection prevention to reduce the risk of SAP and enhance overall prognosis. PNI also serves as a dynamic indicator reflecting changes in elderly sICH patients' nutritional and immune status, guiding individualized treatment and rehabilitation efforts. By continuously monitoring PNI trends, clinicians can promptly evaluate intervention effectiveness and adjust nutritional plans, thereby enabling precision in post‐stroke infection control and nutritional management.

Despite its multicenter design and rigorous statistical analysis, this study has several limitations. First, as a retrospective analysis, it cannot establish causality. Second, it only assessed PNI levels at admission, precluding dynamic observation of how PNI changes during the disease course and influences infection risk. Additionally, all samples were collected from two tertiary hospitals in China, and regional differences may limit the generalizability of the study findings. Furthermore, variations in enrollment timing may influence changes in treatment strategies and diagnostic criteria, thereby potentially affecting the results. The analysis did not adequately adjust for potential confounding factors, such as dietary patterns, drug interventions, antimicrobial strategies, and certain other demographic variables, which may introduce bias. Future prospective cohort studies or randomized controlled trials should further validate the causal relationship between PNI and SAP and investigate the role of WBC as a mediating variable, particularly to verify the potential for reverse causality. Finally, this study lacked analyses of other stroke subtypes, which may limit the generalizability of our findings. Future studies need to conduct prospective subgroup analyses to further explore the situations of these correlations in different types of stroke.

## 5 Conclusions

This study is the first to confirm a significant linear negative relationship between PNI and the risk of SAP in elderly patients with spontaneous intracerebral hemorrhage, revealing partial mediating effects of inflammation. A reduced PNI indicates compromised nutritional and immune function, serving as a critical warning sign for the development of SAP. Moreover, as a computationally simple, low‐cost, and readily accessible clinical indicator, PNI has great potential as a key tool for early risk identification and personalized management in elderly patients with intracerebral hemorrhage. It thus provides a novel theoretical foundation and practical basis for formulating precise nutritional support and infection control strategies.

## Author Contributions

**Wenjun Zhao:** conceptualization, writing – review and editing, writing – original draft, validation. **Boru Hou:** visualization, software, data curation. **Qunbin Shen:** formal analysis, project administration, resources. **Guangyan Li:** conceptualization, methodology, writing – review and editing, writing – original draft. **Dong Wang:** investigation, formal analysis, supervision. **Gang Wang:** writing – review and editing, writing – original draft, funding acquisition, methodology. **JiaYin Wang:** investigation, validation.

## Funding

This study was supported by the Natural Science Foundation of Gansu Province (Grant Numbers: 25JRRA614), University Student Innovation and Entrepreneurship Training Program of Lanzhou University (Grant Number: 20250050020), and Cuiying Scientific Training Program for Undergraduates of The Second Hospital & Clinical Medical School, Lanzhou University (Grant Number: CYXZPT2025‐17).

## Disclosure

The authors declare that no Generative AI was used in the creation of this manuscript.

## Ethics Statement

The study received ethical approval from the Ethics Committees of the Second Affiliated Hospital of Lanzhou University (Approval No.: 2025A‐1073) and the Second Affiliated Hospital of Fujian Medical University (Approval No.: 2024‐016), and strictly adhered to the ethical principles outlined in the Declaration of Helsinki. The need for written informed consent was waived by the Ethics Committee due to the non‐interventional design of the study.

## Consent

All participants consented to publish.

## Conflicts of Interest

The authors declare no conflicts of interest.

## Supporting information


**Table S1:** Analysis of PNI and SAP morbidity.

## Data Availability

The data that support the findings of this study are available from the corresponding author upon reasonable request.
