# Supplementary material for: Relationship Between Prognostic Nutritional Index and Stroke‐Associated Pneumonia in Elderly Patients: A Two‐Center Study of Spontaneous Intracerebral Hemorrhage
Source: Food Sci Nutr. 2026 May 23;14(5):e71941. doi: 10.1002/fsn3.71941 (PMC13240440; doi:10.1002/fsn3.71941)
Supplement: Supplementary file 1 — Table S1: Analysis of PNI and SAP morbidity. [file FSN3-14-e71941-s001.docx]

Supplementary Table 1 Analysis of PNI and SAP Morbidity

| Variables | Total | Non-SAP | SAP | *P*-value |
| --- | --- | --- | --- | --- |
|  | N=431 | N=149 | N=282 |  |
| Age (year) | 71.41 ± 5.2 | 70.95 ± 5.12 | 71.65 ± 5.36 | 0.2 |
| Temperature (℃) | 36.70 ± 1.68 | 36.57 ± 2.78 | 36.76 ± 0.49 | 0.581 |
| Leucocyte (10^9^) | 10.09 ± 4.72 | 8.85 ± 3.80 | 10.75 ± 5.02 | <0.001 |
| Hemoglobin (g/L) | 141.86 ± 22.52 | 147.01 ± 20.18 | 139.13 ± 23.25 | <0.001 |
| Platelets (10^9^) | 191.22 ± 86.19 | 178.49 ± 67.57 | 197.94 ± 93.97 | 0.135 |
| Glucose (mmol/L) | 7.94 ± 2.80 | 7.76 ± 3.13 | 8.04 ± 2.61 | 0.022 |
| PT (s) | 12.37 ± 5.78 | 12.57 ± 4.50 | 12.26 ± 6.35 | 0.907 |
| INR | 1.04 ± 0.28 | 1.08 ± 0.38 | 1.02 ± 0.21 | 0.211 |
| GCS | 10.55 ± 3.42 | 11.94 ± 3.25 | 9.82 ± 3.28 | <0.001 |
| APTT (s) | 26.23 ± 5.98 | 25.97 ± 6.97 | 26.37 ± 5.39 | 0.253 |
| Midline shift (mm) | 2.29 ± 3.69 | 1.12 ± 2.95 | 2.90 ± 3.88 | <0.001 |
| Bleeding volume (mL) | 26.14 ± 22.59 | 20.03 ± 22.91 | 29.37 ± 21.79 | <0.001 |
| PNI | 45.03 ± 5.91 | 45.97 ± 5.63 | 44.53 ± 6.01 | 0.012 |
| Sex, % |  |  |  | 0.246 |
| Female | 190(44.08%) | 60 (40.27%) | 130 (46.10%) |  |
| Male | 241 (55.92%) | 89 (59.73%) | 152 (53.90%) |  |
| History of hypertension, % |  |  |  | 0.042 |
| No | 116(26.91%) | 49 (32.89%) | 67 (23.76%) |  |
| Yes | 315(73.09%) | 100 (67.11%) | 215 (76.24%) |  |
| History of diabetes, % |  |  |  | 0.671 |
| No | 357(82.83%) | 125 (83.89%) | 232 (82.27%) |  |
| Yes | 74 (17.17%) | 24 (16.11%) | 50 (17.73%) |  |
| Smoking history, % |  |  |  | 0.434 |
| No | 356(82.60%) | 126 (84.56%) | 230 (81.56%) |  |
| Yes | 75 (17.40%) | 23 (15.44%) | 52 (18.44%) |  |
| Drinking history, % |  |  |  | 0.764 |
| No | 382(88.63%) | 133 (89.26%) | 249 (88.30%) |  |
| Yes | 49 (11.37%) | 16 (10.74%) | 33 (11.70%) |  |
| History of anticoagulant or antiplatelet drug use, % |  |  |  | 0.601 |
| No | 392(90.95%) | 137 (91.95%) | 255 (90.43%) |  |
| Yes | 39 (9.05%) | 12 (8.05%) | 27 (9.57%) |  |
| Bleeding side, % |  |  |  | 0.173 |
| Bilateral | 1 (0.23%) | 1 (0.67%) | 0 (0.00%) |  |
| Left | 207 (48.03%) | 65 (43.62%) | 142 (50.35%) |  |
| Right | 223(51.74%) | 83 (55.70%) | 140 (49.65%) |  |
| Bleeding location, % |  |  |  | 0.158 |
| Basal Ganglia | 217(50.35%) | 64 (42.95%) | 153 (54.26%) |  |
| Cerebral Cortex | 85 (19.72%) | 35 (23.49%) | 50 (17.73%) |  |
| Cerebellum | 39 (9.05%) | 19 (12.75%) | 20 (7.09%) |  |
| Brainstem | 8 (1.86%) | 2 (1.34%) | 6 (2.13%) |  |
| Thalamus | 27 (6.26%) | 19 (12.75%) | 36 (12.77%) |  |
| Others | 55.00 (12.76%) | 10 (6.71%) | 17 (6.03%) |  |
| Surgical treatment, % |  |  |  | <0.001 |
| No | 259(60.09%) | 122 (81.88%) | 137 (48.58%) |  |
| Yes | 172(39.91%) | 27 (18.12%) | 145 (51.42%) |  |
